# The Benefits of Smart Nanoparticles in Dental Applications

**DOI:** 10.3390/ijms22052585

**Published:** 2021-03-04

**Authors:** Silvia Vasiliu, Stefania Racovita, Ionela Aurica Gugoasa, Maria-Andreea Lungan, Marcel Popa, Jacques Desbrieres

**Affiliations:** 1 “Petru Poni” Institute of Macromolecular Chemistry, Grigore Ghica Voda Alley, No. 41A, 700487 Iasi, Romania; silvia.vasiliu@icmpp.ro; 2 Department of Natural and Synthetic Polymers, Faculty of Chemical Engineering and Environmental Protection, “Gheorghe Asachi” Technical University of Iasi, Prof. Dr. Docent Dimitrie Mangeron Street, No. 73, 700050 Iasi, Romania; ionela.ag21@yahoo.com (I.A.G.); marpopa2001@yahoo.fr (M.P.); 3 Sara Pharm Solutions, Calea Rahovei, No. 226-228, 050909 Bucuresti, Romania; maria_andreea.lungan@yahoo.com; 4 Academy of Romanian Scientists, Splaiul Independentei Street No. 54, 050085 Bucuresti, Romania; 5 Institut des Sciences Analytiques et de Physico-Chimie pour l’Environnement et les Materiaux (IPREM), Pau and Pays de l’Adour University (UPPA), UMR CNRS 5254, Helioparc Pau Pyrenees, 2, av. President Angot, 64053 Pau CEDEX 09, France

**Keywords:** nanoparticles, smart polymers, dental applications

## Abstract

Dentistry, as a branch of medicine, has undergone continuous evolution over time. The scientific world has focused its attention on the development of new methods and materials with improved properties that meet the needs of patients. For this purpose, the replacement of so-called “passive” dental materials that do not interact with the oral environment with “smart/intelligent” materials that have the capability to change their shape, color, or size in response to an externally stimulus, such as the temperature, pH, light, moisture, stress, electric or magnetic fields, and chemical compounds, has received much attention in recent years. A strong trend in dental applications is to apply nanotechnology and smart nanomaterials such as nanoclays, nanofibers, nanocomposites, nanobubbles, nanocapsules, solid-lipid nanoparticles, nanospheres, metallic nanoparticles, nanotubes, and nanocrystals. Among the nanomaterials, the smart nanoparticles present several advantages compared to other materials, creating the possibility to use them in various dental applications, including preventive dentistry, endodontics, restoration, and periodontal diseases. This review is focused on the recent developments and dental applications (drug delivery systems and restoration materials) of smart nanoparticles.

## 1. Introduction

**Figure 1:**
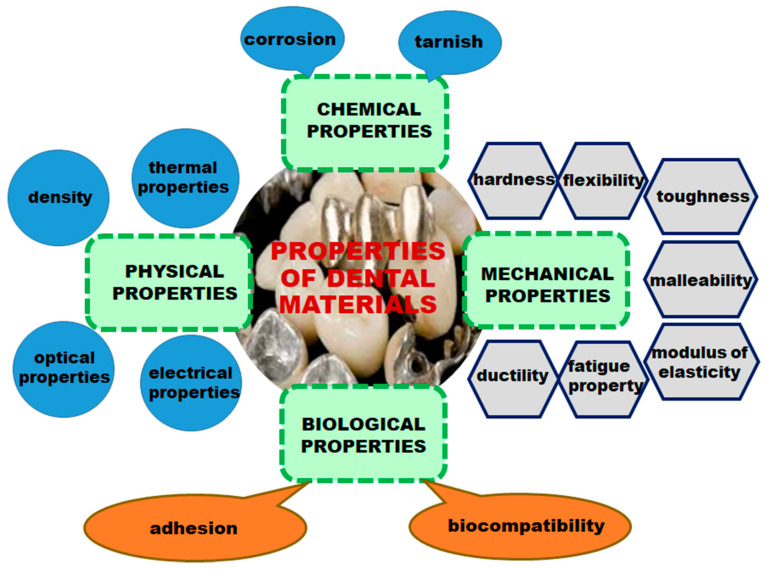
Properties of dental materials.

The evolution of dental materials has passed through several stages, being determined by the complexity of existing technologies at the time. For centuries, the scientific world has constantly sought to find materials that are able to provide properties considered essential for use in oral cavities. For this reason, the materials used in dentistry must meet several physical, chemical, and mechanical requirements (Figure 1), including the following: (1) They must be strong and fracture-resistant; (2) they should be permanently attached to the tooth or bone structure; (3) they should have dimensional stability when exposed to solvents or changes of temperature; (4) they should have a similar appearance to the tooth structure and possess properties similar to tooth enamel and dentin; (5) they should be aesthetic; (6) they should exhibit minimal conduction; (7) the density and abrasion resistance should be close or equal to that of the natural tooth; (8) they should have a sufficient elasticity, low fragility, and transparency; (9) they should be easy to manufacture and process; (10) they should adhere to tissues; (11) they should be tasteless and odorless; (12) they should be easily maintained or fixed; and (13) they should have a reasonable cost [1,2,3,4,5,6].

When choosing a dental material, both mechanical and physical properties, as well as biological properties, must be considered. When a biomaterial is placed in contact with tissues and fluids of the human body, there are several types of interactions between the material and biological environment [7].

In terms of biocompatibility, the dental materials depend on several factors, including the physical and chemical nature of components, the surface characteristics and chemical stability of the material, the type and location of the exposed tissue, and the exposure time [8,9]. Additionally, a material can be said to be biocompatible when it is harmless to soft tissues, it does not contain any toxic substances that diffuse into the circulatory system, it does not contain agents that could lead to an allergic reaction, and it does not contain substances with a carcinogenic potential [10].

Dental materials can be classified as follows:

- Preventive materials, including pit and fissure sealants, sealing agents, liner bases, and cements;
- Restorative materials, including primers, bonding agents, liners, amalgams, composite resin, compomers, hybrid ionomers, ceramics, and metal-ceramics;
- Auxiliary dental materials, including acid-etching solutions, impression materials, casting investment, gypsum, dental waxes, and finishing and polishing abrasives [11].

For a long time, it was believed that so-called “passive” materials are ideal for dental applications because they do not interact with the oral environment. Recently, it has been found that “smart/intelligent” materials that have the ability to modify their shape, color, or size under the action of an external stimulus, such as the temperature, pH, light, moisture, stress, electric or magnetic fields, or chemical compounds, are a good alternative to design materials that can be used in various dental applications.

**Figure 2:**
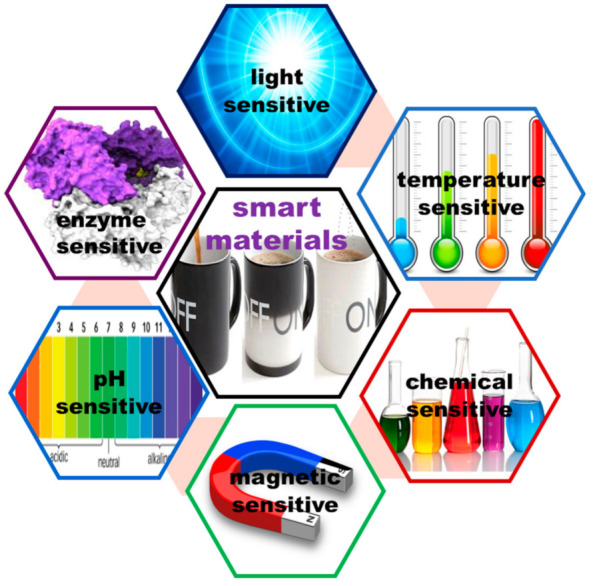
Types of smart materials.

The classification of smart materials is presented in Figure 2 [12].

Another classification divides smart materials into passive materials (glass ionomers cements, resin-modified glass ionomers, compomers, and dental composites) and active materials (smart composites, smart ceramics, smart impression materials, smart burs, shape-memory alloys, smart sutures, and smart antimicrobial peptides). Shanthi and collaborators [13] define passive materials as materials that respond to external changes without external control and also have self-repair characteristics, while active materials detect and respond to changes in the environment.

This review presents information about dental materials and smart dental materials, as well as a history of dental materials, followed by a presentation of the benefits of the use of smart nanoparticles in different dental applications.

## 2. History of Dental Materials

Dentistry, as a branch of medicine, has a long and fascinating history. The first documents on dental problems appeared in ancient Mesopotamia, about 5000 years ago, being engraved on clay tablets with cuneiform writing. However, the first dentists appeared in Egypt in 2600 B.C. and the most important representative was Hesi-Re, also known as “Chief toother” [14]. Tooth forms made of ivory or animal origin were used by different ancient people (Phoenicians, Etruscans, Mayans, and Egyptians) to replace teeth lost in battle, with the Spaniard Alabucasim being the first person to recommend a tooth transplant instead of missing teeth in 1100 B.C. In America, the Mayans and later the Aztecs used semiprecious stones (emerald, turquoise, and jade) for aesthetic purposes. In this operation, they employed both advanced tools and a natural fixing “cement” obtained from a resin mixed with bone meal and various plants. The oldest dentures made of teeth carved from hardwood, ivory, or hippopotamus bone and fixed with gold wire were found among the Phoenicians, while ancient Japanese dentures were made of ebony wood [6].

Additionally, plant fibers, horsehair, silk, or gold and silver wires were used for fixing artificial teeth in the mouth. Originally, the replacement of teeth was only conducted for aesthetics, but over time and with the development of methods for anchoring artificial teeth to natural ones, the problem of improving mastication arose. Much later, Ambroise Pare (1510–1590), an eminent French surgeon, used lead or cork for tooth fillings. After about 100 years, in 1700, M.G. Purmann developed the first models of dental prostheses made of wax [15]. Modern dentistry began in 1728, when Pierre Fauchard published a treatise describing several types of dental restorations, including a new method for the construction of artificial ivory teeth. Then, in the years that followed, a few important steps were observed in the development of both dental materials and dental techniques:

1. The introduction of porcelain (De Chennant, 1789);
2. The discovery of the first dental amalgam in France (Taveau, 1816);
3. The first ceramic tooth (Fonzi, 1808);
4. The first imprint of a root canal (Maggiolo, 1809);
5. The use of rubber and then celluloid for dentures;
6. The first dental “tour” of the foot (Morrison, 1871) and the electric one (Green, 1874);
7. The first substitution crown (Logan, 1884);
8. Black—“the father of modern amalgams”—sets the proportions of alloys (1895);
9. The polymerization of methyl methacrylate—starting point in the preparation of the first acrylic resin for dental use;
10. The burning of ceramics on platinum foil (1936) and in vacuum (1940-1945);
11. The appearance of polyelectrolyte cements as impression materials;
12. The appearance of composite diacrylic resins;
13. The extension of photopolymerization as a polymerization process for various dental materials;
14. The removal of mercury from metal fillings and identification of new solutions;
15. The introduction of titanium in dentistry;
16. The development of smart dental materials as glass ionomer cements, resin-modified ionomer cements, compomers, ormocers, and cermets;
17. The improvement of implantology [16].

In the 20th century, the development of dental techniques has increased the options for tooth treatment. Furthermore, the introduction of controlled drug delivery systems [17,18,19] in dentistry became a necessity, due to the fact that they offer clearly superior advantages over traditional methods, such as the following:

- The drug is released gradually, ensuring a constant concentration in the body at the value corresponding to the therapeutic field;
- The approach helps their metabolism and excretion;
- The side effects of drugs, caused by either overdoses or by their aggression, sometimes to both diseased and healthy cells, are reduced [20].

## 3. Smart Nanoparticles in Dentistry

The introduction of the term nanotechnology by Norio Taniguchi in 1974 [21] represented a huge opportunity for the development of new dental products that could be applied in restorative dentistry, periodontology, implant odontology, biomineralization, oral cancers, endodontics, and adhesive dentistry [22]. Based on their morphologies, nanomaterials with dental applications can be classified as follows: Nanocomposites; nanobubbles; nanocapsules; solid-lipid nanoparticles; nanospheres; metallic nanoparticles; nanotubes; nanocrystals; nanoclays; and nanofibers [23].

The association between the two most frequently employed terms used in the last periods, consisting of “smart” and “nanoparticles”, led to smart nanoparticles, which have received much interest for their uses in the medical field and in particular, in dental applications, including dental implants, polishing of the enamel surface, the prevention of dental cariers, antisensitivity agents, and teeth whitening toothpaste.

The benefits of nanoparticles used in dental applications can be highlighted by their properties:

1. They ensure a large surface area [22,24];
2. They can present antimicrobial, antiviral, and antifungal properties and as a consequence, can prevent biofilm formation when nanoparticles loaded with an antimicrobial agent are incorporated in resin composites [25];
3. They enhance the mechanical properties of dental material, especially in restorative dentistry;
4. They improve the bond strength between dentin and biomaterial;
5. They prevent crack propagation and white spot lesions;
6. They improve the fracture toughness of porcelain restorations [26].

In the literature, two main strategies for the preparation of nanomaterials are depicted: top-down and bottom-up methods. The difference between the techniques consists of the fact that the top-down approach is a destructive method that refers to a decrease in bulk materials to nano-scale particles (mechanical milling, nano-litography (photolitography, electron beam, ion beam, and X-ray litography), laser ablation, sputtering, and thermal decomposition), while the bottom-up approach is a constructive method that refers to the generation of material from atoms to clusters to nanomaterials (chemical vapor deposition, sol-gel method, self-assembly, spinning, pyrolysis, and biosynthesis) [6].

The structural parts of the teeth are as follows:

- Enamel: The very hard, thin, and translucent layer that covers the surface of the dental crown;
- Dentin: A calcified tissue of the tooth situated inside the enamel and cementum;
- Cementum: Specialized calcified substance that is a part of the periodontium and covers the root of a tooth;
- Dental pulp: Unmineralized oral tissue situated in the center of the tooth that is composed of soft connective tissue, blood vessels, lymph vessels, and nervous elements.

### 3.1. Smart Nanoparticles as Drug Delivery Systems

Good oral hygiene is very important for human health because poor oral hygiene can lead to dental diseases, such as gum diseases, infection, bone loss, heart diseases, and strokes.

Millions of bacterial cells are found in the oral environment. Some of them are beneficial, while others are very harmful, causing many oral diseases, such as the following:

1. Bad breath (halitosis) is caused by gum diseases, dental caries, oral cancer, dry mouth, and bacteria on the tongue;
2. Dental caries appear when acids, bacteria, and food form a plaque that covers the teeth [27];
3. Gum diseases (gingivitis and periodontitis) represent an inflammation of the gums, as well as an infection of the tissues caused by the formation of a sticky, colorless plaque on teeth [28];
4. Oral cancers, which include any malignant lesions on the gums, tongue, lips, cheek, floor of the mouth, and hard and soft palate [29];
5. Mouth sores, which are inflammatory disorders characterized by small lesions that develop on the soft tissues in the mouth or at the base of the gums. The following disorders belong to this category: Canker sores (aphtous ulcers); fever blisters or cold sores caused by the *Herpes simplex*; oral thrush or candidiasis; angular cheilosis; fibrous inflammatory hyperplasia; and inflammatory papillary hyperplasia [30];
6. Tooth erosion;
7. Tooth sensitivity;
8. Toothaches and dental emergencies.

As drug delivery systems, smart nanoparticles present superior properties, including a small size; a wider therapeutic window; the ability to overcome multiple drug resistance; multifunctionality; long circulation drugs; the ability to minimize off-target effects; a pH-triggered on/off switchable system; a superior efficacy; a decreased toxicity; improved pharmacokinetics; the feasibility of incorporation of both hydrophilic and hydrophobic active principles; controlled/sustained release; an enhanced permeability and retention; and the feasibility of variable routes of administration, including oral and parenteral administration and inhalation [31,32,33,34].

#### 3.1.1. Periodontal Diseases

One of the most harmful dental disorders, affecting between 5 and 30% of the adult population aged between 25 and 75 years, is periodontitis [35]. Periodontal disease is a disease that is becoming more widespread in modern society and can be a basis for the onset or worsening of diseases such as diabetes, cardiovascular disease, respiratory diseases, cancer, rheumatoid arthritis, and metabolic syndrome.

Periodontal disease is a chronic inflammatory condition caused by the presence of bacterial plaque that leads, in some cases, to the severe destruction of periodontal tissue. Although this disease begins with the accumulation of bacterial plaque, there are a number of favorable factors that can contribute to aggravation of the disease: (a) Local: Smoking, tartar, cavities, and improper nutrition, and (b) general: Physiological conditions (puberty, pregnancy, and menopause); systemic diseases, such as diabetes, leukemia, and anemia; and drug treatments used in epilepsy or cardiovascular diseases.

In the early stages, periodontal disease can be treated by less invasive procedures (scaling, root planning, and antibiotic treatment). At an advanced stage, however, surgical treatment is used, including flap surgery, soft tissue grafts, bone grafts, and assisted tissue regeneration.

This disease exhibits evolution that occurs in three stages: Gingivitis; moderate periodontitis; and severe periodontitis.

Periodontal disease can affect one or more structures of the marginal periodontium: Gums; dental cementum; alveolar bone; and periodontal ligaments. 

In the treatment of periodontal disease, two aspects must be considered: Removal of the biofilm and removal of the bacterial species from dental surfaces [29]. 

*Aggregatibacter actinomycetemcomitans, Porphyromonas gingivalis, Treponema denticola* and *Tannerella* are the bacteria responsible for the triggering of periodontal diseases, but smoking, drinking, stress, and diabetes are other risk factors that determine the occurrence of periodontitis [36].

The standard protocol for the treatment of periodontal disease involves two phases: (1) The mechanical displacement of dental plaque via scaling and root planning, and (2) systemic antibiotic therapy via oral administration [37]. Because systemic antibiotic therapy has several shortcomings, it was necessary to develop the most effective methods that are able to reduce bacterial adhesion and prevent biofilm formation. This approach can be realized by using the nanodrug delivery system in combination with an antimicrobial agent that can be applied directly in the periodontal pocket as a local drug delivery system. 

The success of treating periodontal disease with controlled drug delivery systems depends on their ability to release the antimicrobial agent at the bottom of the periodontal pocket at an appropriate concentration and to facilitate retention of the drug for an appropriate time, which are both necessary for obtaining effective results [38].

In order to treat periodontal disease, in 1979, Goodson and colleagues [39] proposed the first controlled drug delivery system. The field of controlled release systems of biologically active principles is constantly expanding, as evidenced by the fact that researchers continue to develop and improve existing technologies for precise specific release, making adjustments based on the patient’s biological rhythm and disease-specific conditions.

Locally controlled release systems can be classified as follows [40,41]:

1. Depending on the type of treatment application, including personal (treatment of patients at home) and professional (at the dental office), (a) supra- and subgingival irrigation and (b) controlled release systems;
2. According to the biodegradability of the system, biodegradable and non-biodegradable;
3. Depending on the device type, devices with a sustained release of drugs that act in less than 24 h and therefore require multiple applications, and devices with a controlled release of drugs that follow the zero order kinetics and act for a longer period of time.

An ideal drug delivery system for periodontal disease is composed of a biodegradable and bioadhesive carrier-drug system from which the drug is released in a controlled/sustained manner over several weeks [42].

Aminosilane-coated magnetic nanoparticles functionalized with chlorhexidine, which is a second-generation bisguanidine antiseptic, displayed bactericidal and antifungal activities against the microbial biofilm. These smart nanoparticles are recommended for potential use in the treatment of local infections caused by the oral microflora due to the enhanced killing activity in the presence of salivary proteins, as well as the low toxicity against human osteoblasts [43].

A delivery system of tetracycline based on a calcium-deficient hydroxyapatite nanocarrier was obtained for the elimination of infection and bone regeneration. In vitro and in vivo studies revealed that tetracycline-loaded nanoparticles were biocompatible and can serve as osteoconductive bone substitutes with antibacterial and proliferative cell properties [44].

Nanoparticles based on N,N,N-trimethylchitosan, a liposome, and doxycycline showed excellent biocompatibility, inhibited biofilm formation, and prevented alveolar bone resorption, being useful in the treatment of periodontal disease [45].

Another interesting way to treat periodontitis is to formulate an innovative system that has both an antimicrobial and mineralizing effect. For this purpose, calcium phosphate nanoparticles loaded with chlorhexidine were synthesized. To improve the bioadhesivity, the calcium phosphate nanoparticles were coated with carboxymethylcellulose. In this regard, the nanoparticles adhered to the enamel, as well as to the dentin, being able to inhibit the growth of *Escherichia coli* and *Lactobacillus casei* and combat tooth decay and periodontal disease [42,46].

Due to their small size, smart nanoparticles can be administered in periodontal pockets by injection. Injectable systems present an attractive alternative for the release of antibiotics in the periodontal pocket. The application of these systems is extremely easy and fast, considerably reducing the cost of therapy compared to other devices that require time and precision to insert them into the periodontal pocket.

Yang and collaborators [47] prepared an efficient injectable antioxidant defense platform for reactive oxygen species (ROS) removal based on poly(dopamine) nanoparticles as intelligent scavengers, in order to treat oxidative stress-induced periodontal disease. After a post-lingual injection of nanoparticles, the in vitro and in vivo studies revealed that nanoparticles exhibit:

1. Admirable ROS removal;
2. Anti-inflammatory activity;
3. Biodegradable behavior;
4. A high biocompatibility and low systemic toxicity.

Nanoparticles based on poly(D,L-lactide acid) (PLA), poly(glycolic acid), poly(D,L-lactide-co-glycolide acid) (PLGA), and cellulose phthalate loaded with triclosan (TCS), which is a noncationic antimicrobial agent, were prepared by the emulsification-diffusion technique. The TCS-nanoparticles were injected into the bottom of the periodontal pocket and after 15 days, a decrease of inflammation at the experimental sites was observed [23].

Quintanar-Guerrero and collaborators [48] prepared nanospheres and nanocapsules loaded with chlorhexidine using the emulsion-diffusion technique and cellulose acetate phthalate as pH-dependent polymeric material, in order to improve the treatment of periodontitis. These local drug delivery systems can infiltrate or be administered into the periodontal pocket. By applying nanocapsules and nanospheres in the periodontal pocket, a decrease of the dentobacterial plaque index of 65.78% was observed, compared to commercial mouthwash, which reduces this index by only 25.8%. The advantages of using these smart nanoparticles are due to their coverage of a large surface area, the controlled release of drugs at different pH values, a reduction of both the therapeutic dose and treatment time, and potential gingival tissue infiltration.

Chitosan, which is a natural polymer with multiple properties, can be used in the preparation of smart nanoparticles that can be applied in the treatment of periodontal diseases, as follows:

1. Chitosan crosslinked nanoparticles obtained by the emulsion-dispersion technique loaded with sodium fluoride and cetylpyridinium chloride mixed with toothpaste lixivium in order to obtain a product that releases the active substance in a sustained manner [49];
2. Chitosan crosslinked nanoparticles prepared by ionic gelation loaded with sodium fluoride used as dental delivery systems in protection against tooth decay [50];
3. Chitosan-oligonucleotide complexes that are suitable for local therapeutic applications [51];
4. Rose bengal-functionalized chitosan nanoparticles that can present several advantages, including an affinity to bacterial cells and their elimination, increased interaction and uptake caused by the smart nanoparticles, and singlet oxygen release after photoactivation of the photosensitizer (xanthenes with a negative charge) [52].

Lee and collaborators [53] evaluated the potential core-shell poly(D,L-lactide–co-glycolide)-chitosan nanospheres encapsulating simvastatin and doxycycline for the treatment of periodontal disease and large-sized osseous defects, respectively. The core-shell nanospheres with a diameter of 200 nm released the drug sustainably for 28 days. It was observed that the nanospheres promoted the repair of periodontium in periodontitis-affected areas, while at non-infected sites (large-sized mandibular osseous defects), the nanospheres accelerated and augmented osteogenesis.

Chen and collaborators [54] prepared novel pH-responsive quaternary ammonium chitosan-liposome nanoparticles for periodontal treatment. In vivo tests showed that pH-responsive nanoparticles strongly inhibit biofilm formation and prevent alveolar bone absorption, as well as present an excellent biocompatibility with human periodontal ligament fibroblasts. Furthermore, the pH-responsive nanoparticles can be used as a drug delivery system of doxycycline, which is an antibacterial agent, in the treatment of periodontal diseases.

Another study was directed towards the preparation of pH-responsive nanospheres based on PLGA and chitosan as an inflammation-responsive system using the oil in water single emulsion solvent evaporation method. Drugs, metronidazole (an antibiotic), and N-phenacylthiazolium bromide (a host modulator) were encapsulated with PLGA and covered with chitosan in a nanosphere. The studies demonstrated that the subgingival administration of pH-responsive nanospheres encapsulating metronidazole and N-phenacylthiazolium reduced inflammation in the case of periodontitis [55].

#### 3.1.2. Dental Caries

Dental caries is one of the most common and widespread dental diseases [56]. The first classification of dental caries used for many years was realized by G.V. Black (1908) and initially consisted of five categories (Class 1–5), with a sixth being added later (Class 6, Simon’s modification) [57].

Today, based on the knowledge about dental caries accumulated over time, much more elaborate classifications have been developed according to several criteria [58]:

1. The anatomical site: Occlusal; proximal and cervical; linear enamel; and root caries;
2. The type of lesion: Primary and secondary caries;
3. The severity of disease: Acute; chronic; and arrested caries;
4. The extent of caries: Incipient caries; occult caries; and cavitation;
5. The tissue involved: Initial; superficial; moderate; deep; and deep complicate caries;
6. The pathway of caries spread: Forward and backward caries;
7. The number of tooth surfaces involved: Simple; compound; and complex caries;
8. The chronology: Early childhood; adolescent; and adult caries.

Dental plaque formation and the demineralization of teeth are the main factors which cause tooth decay. To solve this problem, antibacterial and remineralization systems were developed. The development of DNA vaccines against *Streptococcus mutans (S. mutans)* represents an effective strategy for preventing the occurrence of tooth decay [36]. Yang and collaborators [59] obtained a new nanoparticle system by incorporating anionic liposomes into chitosan/DNA complexes that can deliver the anti-caries DNA vaccine pGJA-P/Vax into nasal mucosa in a pH-mediated manner. It was observed that the nanoparticles exhibit a higher transfection efficiency, longer residence time, higher level of secretory IgA, longer-term mucosal immunity, and minimal cytotoxicity, and favored clearance via the digestive tract, compared to chitosan-DNA complexes. 

Amorphous calcium phosphate smart nanoparticles prepared by a spray-drying technique could both increase the release of calcium and phosphate ions at a low pH, which is necessary for inhibiting caries formation, and neutralize a lactic acid solution (pH = 4) by increasing the pH to 6, avoiding the formation of caries [60]. Moreover, carbonate hydroxyl apatite nanoparticles are good candidates for biomineralization and repair sizable defects on the tooth surface [61].

#### 3.1.3. Oral Cancer

Cancer is defined as an uncontrolled proliferation of cells inside a tissue or organ. These cells have the ability to replace specific cells in that organ and invade neighboring tissues or organs, causing irreversible damage. Cancer can affect almost any organ or tissue in the body, and the oral cavity is not an exception.

Oral cancer usually appears as an inflammation or lesion on the oral mucosa that does not heal and can occur in several places in the oral cavity, such as the following:

- On the surface, edges, or back of the tongue;
- Inside the cheeks or on the “sky of the mouth”;
- Under the tongue or on the upper or lower lip;
- On the gums or inside the jaws.

Oral cancer is the sixth most common cancer in the world. There are two main causes that lead to this type of cancer, namely, smoking and alcohol consumption [62]. Oral cancer can also be attributed to infections involving viruses such as *Human Papilloma Viruses* (HPV) and *Epstein Barr Virus* [63], fungi such as *Candida albicans* [64], and certain bacteria [65].

Statistical data have shown that over 90% of oral cancers are squamous cell carcinomas [66] that can lead to oral conditions that are potentially malignant. In general, oral cancer discovered at an early stage is curable; however, a delayed diagnosis and rapid metastasis can make treatment extremely difficult [45].

Nanoparticulate materials have unique properties related to their size, morphology, and distribution [67]. Drug-loaded nanoparticle systems can help the immune system (antibodies and cytokines) fight disease at a molecular level [68]. The use of tumor-targeted nanoparticulate systems has the role of both minimizing unwanted side effects and maintaining and improving therapeutic effects [69]. 

One of the most effective cytotoxic agents in the treatment of oral squamous cell carcinoma of the head and neck is cisplatin. The use of cisplatin is limited due to its dose-dependent toxicity, which can affect the ear, gastrointestinal, renal, and neurological systems and blood [70]. To remove these disadvantages, Endo and collaborators [71] synthesized cisplatin-loaded polymeric micelles containing poly(ethylene glycol) (PEG) -poly(glutamic acid) block copolymers. Their studies on mice carrying oral carcinoma showed that cisplatin-loaded nanoparticles had an inhibitory effect equivalent to free drugs, as well as a lower nephrotoxicity.

Recently, synergistic combined treatments have been used to treat squamous cell carcinoma of the head and neck. Therefore, local hyperthermia (HT) is a treatment that, in combination with surgery, radiotherapy and chemotherapy, is used to treat head and neck cancer [72]. New smart nanoparticles have been used in the local HT of head and neck cancer. In particular, noble metal nanoparticles (NPs) are of interest due to localized surface plasmon resonances (LSPRs) resulting from light–matter interactions [73]. However, for the clinical use of NPs, biocompatibility and a prolonged tissue persistence must be taken into account [74]. Therefore, for the use of NPs in oncology, Voliani and collaborators [73] introduced the ultrasmall-in-nano approach to obtain a family of nonpersistent noble metal nano-architectures (NAs). The first multifunctional nano-architecture (tNAs-cisPt) composed of an endogenously double controlled cisplatin prodrug and narrow near infrared-absorbing gold ultrasmall nanoparticles was synthesized by Voliani and collaborators [75]. The antitumor efficacy of tNAs-cisPt was evaluated using three-dimensional (3D) models of squamous cell carcinomas of the head and neck with a different human papillomavirus status. The results obtained show the importance of translatable multifunctional systems, following the design of ultrasmall-in-nano for combined therapeutic applications, such as photodynamic therapy and chemo-radiotherapy [75].

Zhu and collaborators [76] synthesized nanoparticles based on amphiphilic cationic hyperbranched poly(amine-ester) (HPAE). Nanoparticle HPAE is used as a chemotherapeutic system that has the ability to co-deliver both the anticancer drug sodium arsenite (NaAsO_2_) and MutT homolog 1 (MTH1) inhibitor TH287 for the treatment of oral squamous cell carcinoma.

Nowadays, nanotechnology-based gene therapy represents a hope in the modern treatment of cancer [77]. RNA interference (RNAi) is a sequence-specific post-transcriptional gene silencing process in eukaryotes [78]. RNAi can be triggered by microRNA (miRNA) and small interfering RNA (siRNA), which can be designed to target almost any gene [79]. Zhang and collaborators [80] used polyethyleneimine (PEI)-modified magnetic Fe_3_O_4_ nanoparticles to deliver therapeutic siRNAs targeting B-cell lymphoma-2 (BCL2) and Baculoviral IAP repeat-containing 5 (BIRC5) to Ca9-22 oral cancer cells. Studies have shown a higher efficiency of siRNA delivery via Fe_3_O_4_ nanoparticles delivery to Ca9-22 cells than to oral cancer CAL27 cells. These results led to the conclusion that Fe_3_O_4_ nanoparticles may be suitable for siRNA delivery.

An important pathway in cancer therapy is the development of drugs that can be transferred across the cell membrane at concentrations high enough to exert their therapeutic effects inside malignant cells. In this context, viral vectors, dendrimers, liposomes, solid-lipid nanoparticles, and polymeric nanoparticles were investigated for intracellular applications [81,82].

Polymersomes are of particular interest for intracellular drug delivery to oral squamous cell carcinomas. It is known that polymersomes are self-assembled vesicles based on synthetic amphiphilic block copolymers. Studies using polymesomes based on poly-2-(methacryloyloxy) ethyl phosphorylcholine (PMPC) and poly-2-(diisopropylamino) ethyl methacrylate (PDPA) have shown that PMPC-PDPA polymersomes can penetrate and deliver drugs to oral squamous cell carcinomas [83]. These results led to the conclusion that PMPC-PDPA polymers can be used in oral cancer treatment [84].

In the case of oral cancer, chemoprevention can be applied because the oral cavity allows the discovery and observation of the evolution of oral cancer from premalignant oral lesions that have an easily recognizable characteristic appearance. Chemoprevention involves the use of natural or synthetic substances to inhibit, delay, or reverse the carcinogenic process in tissues [85].

In the prevention of oral cancer, three classes of chemopreventive agents are used, namely, vitamin A derivatives (retinoids), non-steroidal anti-inflammatory drugs (NSAIDs, especially cyclooxygenase inhibitors (COX)), and natural products (green tea extract and black raspberries). The therapeutic efficacy of chemopreventive agents is generally improved by the efficiency of the delivery vehicles.

Holpuch and collaborators [86] have conducted a series of investigations for different strategies for the delivery of chemopreventive agents. In one of the works performed by Holpuch and collaborators, they prepared solid lipid nanoparticles with the intention of locally delivering poorly water soluble and unstable agents to human oral tissues. These solid lipid nanoparticles can act as reservoirs of drugs that have the role of both penetrating the basal layer cells of the normal oral epithelium and increasing the amount of drug administered intracellularly into squamous cell carcinomas.

Atanase and collaborators [87] synthesized smart polymeric micelles (PMs) based on poly(2-vinyl pyridine)-b-poly(ethylene oxide) block copolymers as nanocarriers for the encapsulation and controlled release of natural and synthetic hydrophobic drugs. Studies have shown that PMs are sensitive to the pH and protect the natural drug (curcumin) against photodegradation. Curcumin is a natural polyphenolic phytoconstituent used in the pharmaceutical industry due to its anticancer, antioxidant, anti-inflammatory, hyperlipidemic, antibacterial, wound healing, and hepatoprotective properties [88]. Furthermore, in preclinical studies, it has been found that curcumin induces the apoptosis of cancer cells in various tissues or organs [89]. Therefore, these PMs could be used as curcumin delivery vehicles in the prevention of oral cancer.

### 3.2. Smart Materials in Restorative Dentistry

Restorative dental materials are synthetic components used to repair or replace the tooth structure [9]. Dental restoration materials have an ancient history dating back to 600 B.C., when a paste made of silver, tin, and mercury was used for dental restoration in China, being considered the precursor of modern amalgams. A real advance in restorative dentistry was the synthesis of composite diacrylic resins, which revolutionized the working techniques and changed the treatment schemes [9]. Restorative materials can be used for short-time or long-time applications and can be classified as direct restorative materials and indirect restorative materials. Direct tooth restoration represents a simple method in which the bonding materials are placed, shaped, and hardened during a simple procedure. Direct restoration involves the filling of cavities using amalgam, composite resin, glass ionomer cement, resin-modified glass-ionomer cement, compomers, and cermets [90]. Indirect dental restorations require multiple and more complex procedures, including the fabrication of crowns, bridges, dental implants, inlays, onlays, and veneers [91].

The ideal restoration materials must have the following properties: Plasticity—the material must have a plastic setting phase and a plastic working phase; a good chemical-volumetric stability in a sense that it must not expand or contract or dissolve in the mouth; a good bonding strength to the tooth; a low thermal conductivity, in order to stop heat transfer to the pulpal organ; resistance to attrition, abrasion, and chemical erosion; a color that must be identical or close to that of natural teeth; setting time = 3–5 min; a dental ablation process that must be performed as easily as possible; and ease in the handling, application, and finishing of restoration materials [10]. The use of nanotechnology in restorative dentistry presents several advantages, including an improved diametral strength, enhancement of the tensile strength, an increase in the flexural strength, a high fracture toughness, and an increase in the shear bond strength [92]. The nanomaterials used for restorative dentistry are dental nanocomposites, glass-ionomer cements, endodontic sealers, and tooth regeneration nanomaterials [93].

#### 3.2.1. Nanocomposite Resin

Aesthetic and electrochemical problems in the case of amalgams, as well as the fragility, solubility, and low biocompatibility of silicates and PMMA-based resins, led to the development of new materials with improved properties in the 1960s, namely composite resins.

The first commercial types of composite resins, which appeared in the period 1964-1972, quickly entered dental practice. Therefore, due to their exceptional properties, they tend to replace the amalgams, as well as the silicate cements and acrylic resin, used as fillers [94,95,96]. This success was made possible by the following achievements of chemistry and the technology of macromolecular compounds: The synthesis of a large number of diacrylic monomers; the preparation of composites by mixing polymers with different inorganic fillers; and the use of organosilanes as coupling agents for improving the interfacial adhesion of polymer/inorganic surfaces. The use of these discoveries in dentistry to obtain new classes of resins is the undeniable merit of Bowen [97].

**Figure 3:**
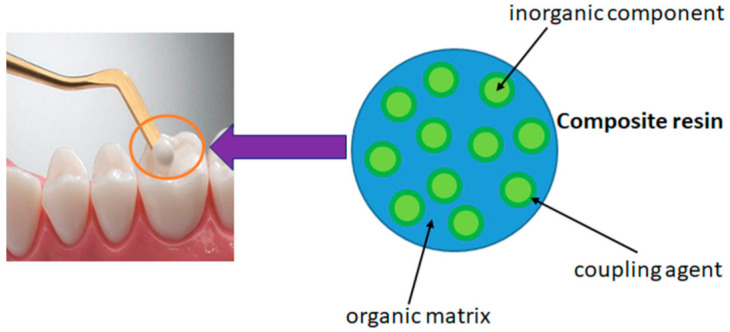
Graphical representation of composite resins.

Composite resins are polymer-based materials that contain the following components: An organic resin matrix (organic phase, 15–30%); filler particles (inorganic phase, 70–85%); a coupling agent (silane); and additives (initiator-accelerator system for polymerization reactions, inhibitors, photosensitizers, UV absorbers, and dyes, 5%) (Figure 3) [98].

**Figure 4:**
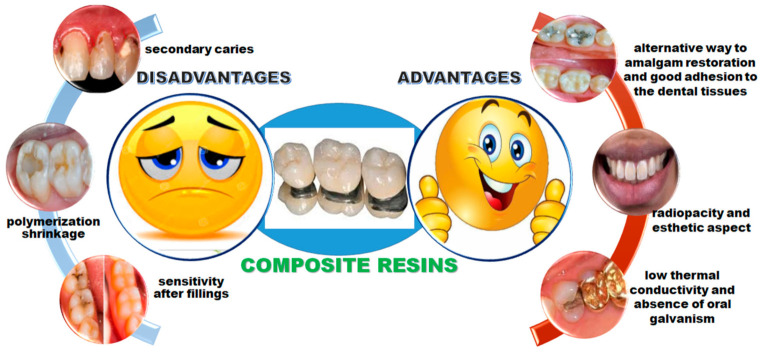
Advantages and disadvantages of composite materials used in dental restoration.

The advantages and disadvantages of composite materials used in dental restoration are presented in Figure 4.

The association between nanoparticles and antimicrobial polycations led to the preparation of a composite resin with improved properties. The nanoparticles can be either physically entrapped within the organic matrix or chemically bound by means of functional groups. The quaternary ammonium polyethyleneimine (QA-PEI) is a polymer that exhibits excellent antibacterial activity [98].

The QA-PEI nanoparticle synthesis follows several steps: (1) A crosslinking reaction in the presence of 1,5 dihalidopentan as a crosslinking agent, resulting in the preparation of crosslinked polyethyleneimine (PEI) nanoparticles; (2) N-alkylation with linear alkyl halides (butyl-hexadecyl alkyl halides); (3) neutralization with sodium bicarbonate; and (4) N-methylation with methyl iodide [99,100].

The antibacterial activity was studied for *Staphylococcus aureus (S. aureus)*, *Staphylococcus epidermitis (S. epidermitis)*, *Enterococcus faecalis (E. faecalis)*, *Pseudomonas aeruginosa (P. aeruginosa)*, and *Escherichia coli (E. coli)*.

It was observed that 2% w/w of incorporated QA-PEI nanoparticles in the organic matrix of the composite resin inhibited the growth of all bacterial strains, while the presence of 1% w/w nanoparticles in the composite resin structure had the effect of the complete inhibition of *S. aureus* and *E. faecalis* and decreased the growth of *S. epidemidis, P. aeruginosa,* and *E. coli* [101].

Additionally, the remineralization of enamel and dentin was enhanced when resin composites containing silver nanoparticles, quaternary ammonium dimethacrylate, or calcium phosphate nanoparticles were used [102,103,104].

#### 3.2.2. Glass-Ionomer Cements

The use of cements in the oral cavity dates back to the pre-Columbian era, being attributed to the Mayan civilization. The first cements, known as zinc phosphate cements, appeared in 1832. Their evolution has continued over time with the development of new types of dental cements, such as zinc silicophosphate (1880), zinc oxide-eugenol (1890), zinc oxysulfate (late 19th century), zinc polycarboxylate (1968, Manchester), ionomers (1972, Wilson and Kent), and resin-modified glass ionomers (1988, Antonucci).

Dental cements are materials that harden based on an acid-base reaction, being used for fixing crowns, bridges, and orthodontic devices, and base and canal fillings. Cements are materials obtained by the reaction between aqueous solutions of carboxylic polyelectrolytes, usually poly(acrylic acid) (PAA) and simple/complex metal oxides or glass ionomers.

The properties of the dental cements must correlate with their clinical use: (1) They should be plastic, so that after preparation, they can be inserted into the cavity or channel; (2) they should not be toxic; (3) they should be insoluble in water; (4) the thermal expansion should be approximately the same as that of hard dental tissues; (5) they should have a good compressive, tensile, and flexural strength; and (6) they should not suffer from contractions [105].

So far, the following types of dental materials based on polyelectrolytes have been obtained:

- Zinc polycarboxylate (ZPC) cements described by Smith, with zinc oxide being the cation source;
- The ionomer cements (GIC) described by Wilson and Kent, with fluoroaluminosilicate glass being the cation source;
- Alginate-based impression materials [106].

In 1969, Wilson and Kent [107,108] replaced ZnO with fluoroaluminosilicate glass, developing so-called glass ionomer cements.

Glass ionomer cements are formed by the acid-base reaction between an aqueous solution (approximately 50%) of polyacid acting as a proton donor and a finely powdered fluoroaluminosilicate glass (20–50 μm) acting as a proton acceptor. The poly(acrylic acid) or copolymers of acrylic, itaconic, or maleic acids are the most common polyacids used in the preparation of glass ionomer cements. A small amount of (+) tartaric acid is also added to the acid solution and is used as a reaction control agent [109].

**Figure 5:**
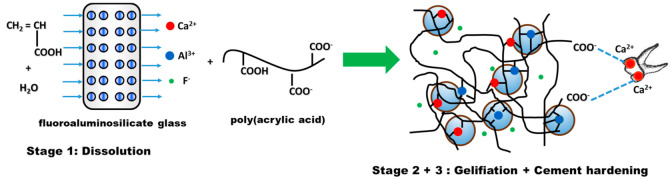
Setting reaction of glass ionomer cements.

The setting reaction of glass ionomers presents three stages (Figure 5):

- Stage 1—Dissolution, which consists of mixing water-soluble polyacid with fine fluoroaluminosilicate glass powder [110]. Due to PAA ionization, hydrogen ions are released and attack the surface layer of glass powder, while the glass interior remains intact and will act as a filler in the cement matrix. The attack of the acid on the glass particles has the effect of first releasing the Ca^2+^ ions and then the Al^3+^ ones;
- Stage 2—Gelling. The released cations react with the carboxylate groups belonging to the polyacid chains, leading to the formation of ionic crosslinks, which result in the development of a hydrogel-type network around the glass particles. In this phase, water plays a very important role. If, in the first stage, the water is totally incorporated into the structure of the cement, during the gelling reaction, the cement paste must be protected from the addition of water, in order to prevent negative consequences for the formation of the cement [111];
- Stage 3—Cement hardening is a process that can take longer. In general, it is necessary for about 30 min for the reaction between Al ions and the reactive groups situated on the polyelectrolyte chain to take place [112]. The ionic crosslinking process is responsible for the hardening of the ionomer cement. Studies conducted by Wasson and Nicholson [113,114,115] suggest that dissolution of the glass fractions is achieved and the silicate network is responsible for the fragmentation of the cementation. This hypothesis is based on the fact that glass powders and acetic acid form cements after one week and their strength increases after a period of 6 months.

The powder–liquid ratio and the composition of the glass phase have a strong effect on the kinetics of the preparation reaction and the properties of glass ionomer cements. The system most widely used as a proton acceptor is fluoroaluminosilicate glass, containing silica (25–35% SiO_2_), alumina (14–20% Al_2_O_3_), calcium fluoride (13–35% CaF_2_), sodium fluoride (4–6% NaF_3_), aluminum fluoride (4–6% AlF_3_), aluminum phosphate (10–25% AlPO_4_), and cryolite (5–20% Na_3_AlF_6_) [116]. The fluoride present in this type of glass plays an important role in the cariostatic properties of dental cements, changes the refractive index, and helps to obtain transparent cements. Fluoride is also responsible for destruction of the glass network and at the same time, for the increase of the susceptibility of glass to acid degradation. The strength and reactivity of the glass, the working and setting times, and the optical clarity are affected by changes of the glass composition. Furthermore, the effects of environmental conditions on the hardness and elasticity modulus of ionomer glass cements were investigated. The results suggested that the mechanical properties of GIC are typical to the restorative materials and are dependent on the storage conditions. Therefore, the oral environment is very important for the clinical selection of GIC.

To improve various properties of GIC, research was directed towards the modification of glass ionomer cement by the introduction of apatite nanoparticles [117] or by the inclusion of different types of nanoionomers [118].

The incorporation of nanohydroxy and fluorapatite nanoparticles synthesized by the sol-gel technique into commercial glass ionomer powder led to an improvement of the biological and mechanical properties (compressive strength, diametral tensile, hardness, and biaxial flexural strength) of new smart cements compared to commercial GICs.

When TiO_2_ nanoparticles were incorporated into GIC, an improvement of the mechanical properties and antibacterial activity against *Streptococcus mutans* was observed [116].

In order to improve the mechanical properties without affecting the clinical properties, Rehman and collaborators [119] modified the conventional glass ionomer (Fuji II, GC International, Tokyo, Japan) with N-vinylpyrrolidone (NVP) containing polyacids, as well as with nano-hydroxy and fluoroapatite. The synthesized copolymers based on acrylic acid and N-vinylpyrrolidone with side chains containing itaconic acid were used in glass-ionomer cement formulation. The nanoceramic particles (nano-hydroxy or fluoroapatite) were synthesized using the ethanol-based sol-gel technique. These nanoparticles were also incorporated in commercial glass ionomer Fuji II. The addition of NVP, nano-hydrohyapatite, and nanofluoroapatite into glass ionomer cement led to an enhancement of the mechanical strength compared to commercial Fuji II glass ionomer cement.

Glass ionomers are widely used in restorations of anterior teeth, as fillers for cavities, in cementations, and as adhesive materials for attaching metal and synthetic restorations. GICs exhibit a low exothermic reaction, and offer a pleasant appearance, good marginal seal, and very good biological properties due to the sustained release of fluoride ions, which accumulate in neighboring areas of enamel and dentin, reducing the risk of secondary caries and side effects [120].

In order to improve some shortcomings, the current research has focused on obtaining new smart materials with complex structures, such as the following:

1. Hybrid materials—GIC modified by the addition of resin (resin-modified ionomer cement);
2. Composites modified by the addition of polyacid (compomers);
3. Composites modified by the coupling of an inorganic network to the organic matrix (ormocers);
4. GIC with the addition of modified resins by incorporating prepolymerized particles (giomers).

#### 3.2.3. Resin-Modified Glass Ionomer Cements

The introduction of the first glass ionomer/resin composite hybrid material known as resin-modified glass ionomer cements (RMGICs) in 1990 by Mathis and Ferracane [121,122] was an attempt to solve several shortcomings caused by the GIC. Therefore, cements were developed by light curing resin-modified glass ionomers with the following advantages: Ease of handling during work; the formation of strong bonds; stability over time and with temperature change; resistance to dehydration; a longer working time; a shorter setting time; the possibility of immediate finishing; high fluoride release; highly radiopaque; a high translucency for better aesthetic results; and biocompatibility. The composition of these cements consists of a polyelectrolyte solution, a hydroxyethylmethacrylate (HEMA)/water mixture, and a small amount of photoinitiator used for the polymerization of HEMA. When this liquid is mixed with glass powder, the setting reaction rate is slowed down due to the low water content, thus increasing the working time. When placed in the mouth and under UV light, the cement hardens quickly and HEMA is polymerized.

The mechanism for the preparation of RMGICs consists of three steps: (a) An acid-base reaction between the poly(acrylic acid) and the fluoroaluminosilicate glass; (b) a photo-initiated free-radical reaction between methacrylate monomers; and (c) a chemically-initiated reaction between methacrylate monomers remaining after photoinitiation [123,124].

Light-curable GIC is resistant to water contamination and does not require protective varnish. The composition of the cements can be modified by the addition of hydroxyacrylates, Bis-GMA, and a small amount of PAA, which is able to copolymerize with HEMA. GIC modified with resins generally presents a strong adhesion to enamel and dentin, and when exposed to an aqueous environment, can release F, Ca, Na, or Al ions [125,126]. Initially, these types of cements were obtained by using them as liners, but later, they were used in base fillings and cementations [127,128]. However, it was found that, after 24 h, HEMA may diffuse through the dentin and affect the pulp, thus increasing the cytotoxicity of the dental material [129].

For this reason, resin-modified glass ionomers were obtained without the addition of HEMA in the composition. Therefore, Xie and collaborators [130] obtained light-curable GICs that are biocompatible and possess improved mechanical properties compared to the commercial cement Fuji II LC type. The process of obtaining light-curable GIC involves two stages:

1. The synthesis of acrylic and methacrylic derivatives of amino acids, such as aspartic acid, glutamic acid, and α-alanine via the Schotten–Baumann reaction [131], and
2. The copolymerization of monomers containing acryloyl and methacryloyl groups with acid acrylic.

**Figure 6:**
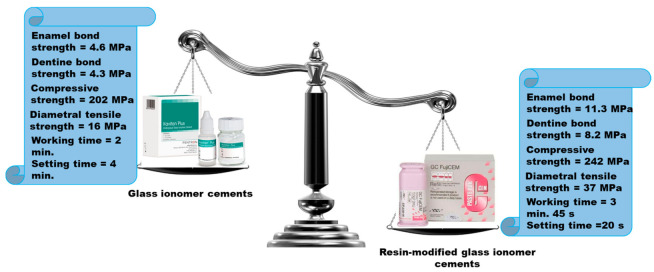
Properties of resin-modified glass ionomers compared with glass ionomers.

The properties of resin-modified glass ionomers are presented in Figure 6.

The incorporation of nanoparticles into RMGICs by the 3M ESPE Company led to the preparation of a new category of restorative materials, called nanoionomers.

The nanoionomers contain copolymers of acrylic and itaconic acid, alumino-silicate glass, bisphenol A-glycidyl methacrylate, triethyleneglycol dimethacrylate, hydroxyethyl methacrylate, and nanofillers.

In vitro studies have shown that the nanofillers provide enhanced wear and polish, as well as a low hardness value, compared to some commercial cements. However, the advantages of this dental nanomaterial is that it provides enhanced aesthetics, fluoride release, and the ability to create a caries inhibition zone after acid exposure [132,133].

Resin-modified glass ionomers are used in various clinical applications, such as temporary fillings on permanent teeth, liners or bases, Class II and III direct composite restorations known as the “sandwich technique” [134], sealants, dentin substitutes, primarily cervical fillings, and root caries.

## 4. Commercial Dental Nanomaterials

**Figure 7:**
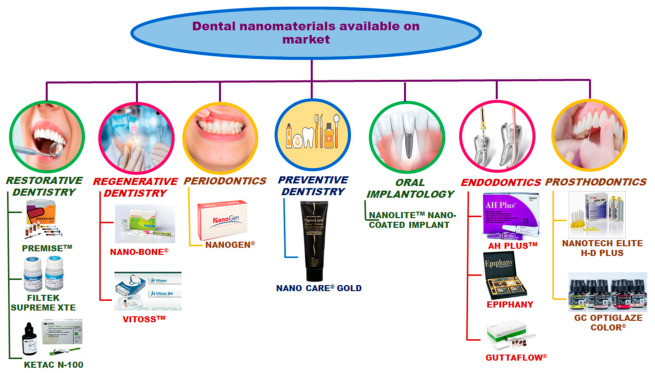
Dental nanomaterials available on the market.

Some commercial dental nanomaterials are presented in Figure 7.

3M ESPE, St. Paul, MN, USA is a company that has a long-standing history (more than 100 years) in the development of high-quality, innovative products for dental applications.

Two outstanding dental nanomaterials produced by 3M ESPE Company (hybrid nanocomposite Filtek Supreme XT and Ketac^TM^ Nano Light Curing Glass Ionomer Restorative) are leaders in the dental materials market. Filtek Supreme XT Universal Restorative is a visible-light-activated hybrid nanocomposite that contains bisphenol A-glycidyl methacrylate (bis-GMA), urethane-dimetahcrylate (UDMA), triethylene glycol dimethacrylate (TEGDMA), and bisphenol A polyethylene glycol diether dimethacrylate (bis-EMA resin(6)). The filler is represented by a combination of non-agglomerated/non-aggregated 20 nm silica filler, non-agglomerated/no aggregated 4 to 11 nm zirconia filler, and aggregated zirconia/silica cluster filler. This hybrid nanocomposite is designed for use in anterior and posterior restorations and presents the following characteristics: It is versatile; it displays exceptional handling; it is naturally aesthetic; it is an excellent polish; it exhibits an improved fluorescence; and it is a unique nanofiller technology [135].

Ketac N-100 is the first resin-modified glass ionomer cement designed based on nano-filler technology. Ketac N-100 nanoionomer represents a combination of fluoraluminosilicate technology and the concept of nanotechnology encountered in Filtek^TM^ Supreme Universal Restorative preparation. By using nano-fillers and nanoclusters, as well as fluoraluminosilicate glass, a number of improvements have been observed:

1. Excellent aesthetics;
2. High initial polish;
3. High fluoride release that is rechargeable;
4. Ability to create a caries inhibition zone after acid exposure;
5. Higher wear resistance;
6. Fast and ease to handle.

This nanoionomer is used in primary teeth and transitional restorations; small Class I, as well as Class III and IV, restorations; sandwich restorations; and core build-ups [136].

Fuji IX GP is a smart dental nanomaterial that mimics the behavior of human dentin and contains a next generation of glass filler, namely SmartGlass^TM^. The main characteristics of this restoration nanomaterial are a higher translucency, fluoride release, reactivity, and a faster setting time [137,138]. The natural properties of the glass ionomer in combination with exceptional handling lead to the conclusion that Fuji IX GP meets all of the requirements of modern restoration materials.

Nanotech Elite H-D+ (Zhermack, Badia Polesine, Italy) is an impression material that combines the hydrocompatibility with nanotechnology concept in order to improve the fluidity and enhance detail reproduction [139].

## 5. Nanotoxicity

A nanomaterial is defined as a structure possessing, at the minimum, one external dimension measuring 1–100 nm, and has a specific property or composition [140].

Nanotoxicology is focused on the study of the adverse effects of nanomaterials on human health and the environment [141]. The entry routes of natural and anthropogenic nanoparticles in the body are human skin, lungs, the gastrointestinal tract, implants, and injections. The toxicity of the nanoparticles depends on different factors, such as the exposure time, particle size, aggregation, concentration, surface area, crystallinity, surface functionalization, individual genetic complement, etc. [142].

Due to their small size, nanoparticles can have many beneficial health effects, but they can also have a detrimental influence on the human body:

1. They can produce irreversible damage to cells by oxidative stress;
2. They can influence basic cellular processes;
3. They can cause an inflammatory response.

There are only a few studies in the literature on the toxicity of nanoparticles in dental applications. Casarin and collaborators [143] evaluated the clinical, microbiological, and immunological performance of PLGA nanospheres encapsulating 20% doxycycline as adjunctive therapy in chronic periodontitis in type-2 diabetic subjects. This study was realized on 40 patients with type-2 diabetes mellitus and chronic periodontitis as a parallel, double blind, randomized, and placebo-controlled clinical trial. The local application of PLGA-doxycycline nanospheres in the deep periodontal pocket favored cytokine modulation and periodontal pathogen level reduction, leading to the conclusion that the nanospheres can be used as adjuvants in the therapy of periodontitis in type-2 diabetic patients.

The new doxycycline loaded nanoparticles based on chitosan and carboxymethylchitosan have a stable structure and bacteriostatic ability against *Porphyromonas gingivalis*. Additionally, the drug-nanoparticle systems were characterized by an orderly morphology and excellent cytocompatibility. These systems down-regulated both the gene and protein levels of NLRP3 inflammasome and IL-1β in HGF_s_ [144]. The NLRP3 inflammasome is responsible for regulating innate immune responses in chronic inflammatory diseases and NLRP3 inflammasome inhibition is a new target in the treatment of periodontal disease [145].

## 6. Future Perspectives

Dentistry has exhibited extensive evolution over the time. The development of new dental materials and techniques for treating dental problems has been the concern of scientists from this field. 

Nowadays, the materials obtained at a nanoscale have revolutionized the scientific world, but understanding the benefits of “active” nanomaterials versus “passive” nanomaterials can open up many opportunities for the development of smart nanomaterials that can be applied in dental applications, including nanodiagnosis (diagnosis of cancer), nanoprevention (sealants, nanosilver fluorides, and nanohydroxyapatite toothpaste), and nanotreatment (endodontics, restorative treatment, surgical treatment, and periodontal diseases). The combination of smart nanoparticles and protein and gene delivery nanotechnologies or photodynamic therapy represents the future trend in dentistry. Moreover, the incorporation of smart nanoparticles that exhibit antibacterial activity in the composition of hybrid and composite dental materials can ameliorate or eradicate gum diseases. The development of nanoscale devices that can be used for diagnosis, orthodontic treatment, or local anesthesia represents a challenge for future researchers. A few steps in the nanoscale devices field have already been taken, but more and deep investigations and clinical trials are needed for the application of nanotechnology, especially nanoscale devices, to solve several dental problems. A very important issue is elucidating the toxicological aspect of nanoparticles because this problem has not been sufficiently studied.

In conclusion, the stimuli responsive or self-repairing nanomaterials and nanomaterials for dental hard tissue regeneration can represent solutions to simple and complex dental problems, and may represent an alternative for obtaining the next generation of dental materials.
